# Sulforaphane Delays Intervertebral Disc Degeneration by Alleviating Endoplasmic Reticulum Stress in Nucleus Pulposus Cells via Activating Nrf-2/HO-1

**DOI:** 10.1155/2023/3626091

**Published:** 2023-01-07

**Authors:** Xiao Lu, Guangyu Xu, Zhidi Lin, Jian Song, Yuxuan Zhang, Hongli Wang, Feizhou Lu, Xinlei Xia, Xiaosheng Ma, Fei Zou, Jianyuan Jiang

**Affiliations:** Department of Orthopedics, Huashan Hospital, Fudan University, No. 12, Middle Wulumuqi Road, Jing'an District, Shanghai 200040, China

## Abstract

Intervertebral disc degeneration (IVDD) is one of the main causes of low back pain, which brings heavy burdens to individuals and society. The mechanism of IVDD is complex and diverse. One of the important reasons is that the abnormal accumulation of reactive oxygen species (ROS) in nucleus pulposus cells (NPCs) leads to endoplasmic reticulum stress (ERS), which causes increased apoptosis of NPCs. Nuclear factor E2-related factor 2 (Nrf-2) and its downstream antioxidant proteins are key molecular switches for sensing oxidative stress and regulating antioxidant responses in cells. Sulforaphane (SFN), a natural compound derived from *Brassicaceae* plants, is a Nrf-2 agonist that displays potent antioxidant potential in vitro and in vivo. Here, we used advanced glycation end products (AGEs) to construct an in vitro degeneration model of NPCs, and we found that AGEs elevated ROS level in NPCs and caused severe ERS and apoptosis. While SFN can promote the entry of Nrf-2 into the nucleus and increase the expression level of heme oxygenase 1 (HO-1) in vitro, thus clearing the accumulated ROS in cells and alleviating ERS and cell apoptosis. Moreover, the protection of SFN on NPCs was greatly attenuated after HO-1 was inhibited. We also used AGEs to construct a rat IVDD model. Consistent with the in vitro experiments, SFN could attenuate ERS in NPCs in vivo and delay disc degeneration in rats. This study found that SFN can be used as a new and promising agent for the treatment of IVDD.

## 1. Introduction

Low back pain (LBP) is a common disease with high rate of incidence and wide coverage in modern population [1]. With the development of society and changes in people's lifestyles, the incidence of lower back pain is increasing year by year [2, 3], and the patients tend to be younger [4]. Data show that about 84% of the world's population suffers from LBP, of which 10% will be disabled [5, 6]. Therefore, LBP not only seriously influences patients' physical and mental health and quality of life but also causes a heavy economic burden on families and society [7]. In 2016 alone, medical care related to LBP cost the United States as much as 134.5 billion dollars [8]. A large number of studies have proved that intervertebral disc (IVD) degeneration (IVDD) is the main cause of LBP [9–11].

IVDD is mainly characterized by the decrease of nucleus pulposus (NP) cells (NPCs) and the degradation of extracellular matrix (ECM). NPCs are the main source of ECM, but the pathophysiological mechanism of the reduction in the number of NPCs is not fully understood [12]. Endoplasmic reticulum (ER) plays a fundamental role in regulating the normal physiological function of NPCs by virtue of its strong membrane structure and a large number of enzymes on it [13]. ER stress (ERS) is a series of adaptive cellular responses when ER homeostasis is destroyed. When ERS occurs, the unfolded protein response (UPR) is activated with the goal of protecting the cell from stress, reducing biosynthetic load, and helping reestablish cellular homeostasis [14]. But, persistent ERS would further aggravate the pressure of ER and even induce cell death through UPR signaling pathway.

Excessive oxidative stress, which causes accumulation of reactive oxygen species (ROS), is one of the common causes of ERS [15]. Nuclear factor E2-related factor 2 (Nrf-2) is a vital molecular switch for sensing oxidative stress and regulating antioxidant responses. Under oxidative stress, Nrf-2 is activated and translocated into the nucleus, regulates the expression of many downstream antioxidant proteins including heme oxygenase 1 (HO-1), which protects against cellular oxidative stress injury and exerts cytoprotective effects [16, 17].

In recent years, natural plant-derived compounds have been extensively studied for the treatment of IVDD [18]. Sulforaphane (SFN) is widely found in the *Brassicaceae* family and is most abundant in broccoli. A recent study showed that SFN exhibited antiviral activity against pandemic SARS-CoV-2 and seasonal HCoV-OC43 coronaviruses [19]. Besides, it has pharmacological effects such as antioxidation, antitumor, antibacterial, and anti-inflammatory [20–22]. And its antioxidant capacity is mainly achieved through the activation of Nrf-2.

In this study, we found that the level of ERS was higher in the degenerated NP tissues. By constructing a cell model of oxidative stress with advanced glycation end products (AGEs), we found that SFN could activate the Nrf-2/HO-1 pathway to alleviate ERS and apoptosis in NPCs. In addition, animal experiments have also demonstrated that SFN has the effect of ameliorating IVDD. This study provided a new and promising protocol for the treatment of IVDD.

## 2. Materials and Methods

### 2.1. Ethics Statement

Acquisition of human nucleus pulposus tissue was approved by the ethics committee of Huashan Hospital, Fudan University (No. KY2022-044). The conduct of animal experiments was approved by the animal ethics committee of Shanghai Medical College, Fudan University (No. 202203012S).

### 2.2. NPCs Isolation and Culture

IVD is divided into five grades according to the Pfirrmann grading [23]. We divided grades I to III into negative control (NC) group and grades IV to V into IVDD group. The NC group NP tissues were obtained from patients with Hirayama disease because they hardly undergo degeneration. The NP tissues in the IVDD group were derived from patients with cervical myelopathy, and they tended to degenerate severely. Fifteen samples were used for WB and 30 samples were used for qRT-PCR. Samples from five NC groups were used for cell culture. NP tissues were cut, separated by 0.25% trypsin (Gibco, US), and incubated in DMEM/F12 complete medium (Gibco, US). The supernatant was discarded by centrifugation, 0.2% collagenase type II was added, and centrifuged for 3 h until the tissue pieces disappeared. The cells were inoculated into the culture bottle and the culture medium was changed once every 3 days. After forming monolayers, they were detached with 0.25% trypsin and passaged. Cells used in experiments were all passaged until passages 1 to 3.

### 2.3. Cell Grouping and Treatment

Controls were treated without any treatment. SFN group was as follows: the cells were treated with 10 *μ*M SFN for 24 hours. AGEs group was as follows: the cells were treated with 200 *μ*g/mL AGEs for 24 hours. SFN + AGEs group was as follows: the cells were treated with 10 *μ*M SFN and 200 *μ*g/mL AGEs for 24 hours. SFN + AGEs+ML385 group was as follows: the cells were treated with 10 *μ*M SFN, 200 *μ*g/mL AGEs, and 5 *μ*M ML385 for 24 hours. SFN + AGEs+si NC group was as follows: the cells were treated with 10 *μ*M SFN, 200 *μ*g/mL AGEs, and si NC for 24 hours. SFN + AGEs+si HO-1 group was as follows: the cells were treated with 10 *μ*M SFN, 200 *μ*g/mL AGEs, and si HO-1 for 24 hours. All NPCs were cultured under 1% hypoxia to simulate the hypoxia environment of intervertebral disc in vivo.

### 2.4. Western Blot (WB)

Total protein was extracted using RIPA kit (AS1004, ASPEN), and protease inhibitors (AS1008, ASPEN) were added during protein extraction to prevent protein degradation. After electrophoresis, coating and sealing, the indexes to be detected were incubated with primary antibody and secondary antibody, and finally immunolabeled with enhanced chemiluminescence reagent (AS1059, ASPEN). Antibodies against caspase-3 (#9662), caspase-12 (#9671), phosphorylated PERK (p-PERK, Thr980, #3179), PERK (#3192), phosphorylated eIF2*α* (p-eIF2*α*, Ser51, #3597), eIF2*α* (#2103), GRP78 (#3183), CHOP (#2895), and HO-1 (#43966) were obtained from cell signing technology corporation (Massachusetts, USA). Anti-*β*-tubulin (#ab6046), anti-*β*-actin (#ab8226), and anti-Nrf-2 (#ab137550) were purchased from Abcam (Cambridge, USA). Antihistone H3 (EM1108) was purchased from ELK Biotechnology (Wuhan, China).

### 2.5. Quantitative Real-Time Polymerase Chain Reaction (qRT-PCR)

TRIpure reagent (EP013, ELK Biotechnology) was used to extract total RNA from NP tissues and cultured cells. The primers used for qRT-PCR were as follows: homo GRP78, forward 5′-CATCACGCCGTCCTATGTCG-3′, and reverse 5′-CGTCAAAGACCGTGTTCTCG-3′. Homo CHOP, forward 5′-CCCTCACTCTCCAGATTCCAGTC-3′, and reverse 5′-CTAGCTGTGCCACTTTCCTTTCA-3′. Homo GAPDH, forward 5′-TCAAGAAGGTGGTGAAGCAGG-3′, and reverse 5′-TCAAAGGTGGAGGAGTGGGT-3′. GAPDH was used for normalization.

### 2.6. Immunofluorescence Staining

After drying the sections naturally, they were rinsed with 1 × PBS and washed 3 times for 5 min each. The antigen repair solution is 10 mmol/L Tris EDTA (pH 9.0) (V900483, Sigma). After the repair, when it returns to room temperature, clean the sections again. After blocking with 10% goat serum for 1 hour, the primary antibody (concentration 1 : 200) was incubated overnight at 4°C. Next day, the slices were washed with 1 × PBS for 3 times for 5 min each time, and then incubated with fluorescent secondary antibody (concentration 1 : 200) for 1 h and 4-6-diamidino-2-phenylindole (DAPI) (D8417-1MG, Sigma) for 5 min. After incubation, cleaned the sections, sealed them with antifluorescence quenching agent (V900155-25G, Sigma), and observed and took photos after natural drying in dark.

### 2.7. TdT-Mediated dUTP Nick End Labeling (TUNEL) Staining

After the cells were inoculated into 12-well plates for intervention treatment, they were washed with PBS once, fixed with 4% paraformaldehyde (80096618, Sinopharm Chemical Reagent) at 37°C for 30 minutes, washed with PBS three times, and then treated with 0.1% Triton X-100 (30188928, Sinopharm Chemical Reagent) for 5 minutes. After washing with PBS three times, 50 *μ*L TUNEL solution (11684817910, Roche) were added, respectively, incubated in dark at 37°C for 60 min, washed with PBS, added antifluorescence quenching sealing solution (V900155-25G, Sigma), placed it in 40 times fluorescence microscope, randomly selected 10 fields, counted the number of TUNEL positive cells and total cells, observed and took photos.

### 2.8. Flow Cytometry

After the cells were centrifuged for 5 min at room temperature and resuspended in PBS at 4°C and washed by centrifugation, they were suspended in binding buffer diluted with deionized water and incubated for 15 min at room temperature in the dark by adding annexin V-FITC (AO2001-02P-G, Sungene Biotech) to the PI label, and the diluted binding buffer was added and then used for detection on the machine (AriaIII, BD).

### 2.9. Transfection of Small Interfering RNA (siRNA)

Inhibition of HO-1 expression in NPCs was performed using small interfering RNA (siRNA). The sequences of si-RNAs for HO-1 were as follows: sense: 5′-CAGAUCAGCACUAGCUCAUTT-3′; antisense: 5′-AUGAGCUAGUGCUGAUCUGTT-3′. NPCs were digested with 0.25% trypsin and counted and inoculated into 6-well plates. Lipofectamine 2000 and trail siRNA DMEM were added, and the final concentration was 200 nmol/L. Knockdown efficacy was examined by WB.

### 2.10. Detection of Reactive Oxygen Species (ROS)

CellROX Deep Red Reagent (#C10422, Thermo Fisher Scientific) was added to the culture medium and incubated for 20 minutes. A fluorescence microscope was used to observe the cells staining situation, and five visual fields were randomly selected. The intensity of red fluorescence per unit area was analyzed by Image Pro Plus 5.0 image analysis system, and the relative content of ROS in cells was expressed by the intensity of fluorescence per unit area.

### 2.11. Extraction of Cytoplasmic and Nuclear Protein

After treatment, cytosolic and nuclear proteins were detached by using the Nuclear and Cytoplasmic Protein Extraction Kit (#P0027, Beyotime Biotechnology). After protein quantification, the variation of Nrf-2 expression was detected via WB.

### 2.12. Animal Experiments

A total of 30 six-week-old Sprague-Dawley (SD) rats were used for the following animal experiments. The rats were randomly divided into three groups, the control (PBS) group (ten females), AGEs group (ten females), and AGEs+SFN group (ten females). All rats were anaesthetized by intraperitoneal injection of a mixture of 70 mg/kg ketamine (Hengrui, China) and 5 mg/kg xylazine [24]. Co8/9 was selected for intradiscal injection of PBS, advanced glycation end products (AGEs) (200 *μ*g/mL) or a mixture of AGEs (200 *μ*g/mL) + SFN (10 *μ*mol/L), respectively, at the same total injection volume (2 *μ*L), using a 33-gauge needle (Hamilton, Benade, Switzerland) [13]. Intradiscal injections were performed every 2 weeks, and rats were housed for 8 weeks. A 33 gauge needle can avoid intervertebral disc degeneration caused by acupuncture to the greatest extent.

### 2.13. MR Examination of Tail Vertebrae

The tail vertebrae of rats were examined by MR (3.0 T, Prism, Siemens, Germany) at 0, 4, and 8 weeks, respectively. Degenerative discs show reduced brightness and volume. After 8 weeks, the intervertebral discs were scored by the Pfirrmann grading according to the previous research methods [25].

### 2.14. Histology and Immunohistochemistry

Rats were sacrificed 8 weeks later, and tails were collected. The fur and muscle of the tail were fully removed, fixed with 4% paraformaldehyde, and then decalcified with EDTA. After decalcification, dehydration and paraffin embedding were carried out, and then dewaxing, hematoxylin and eosin (H&E) staining, and Safranin-O/fast green staining were carried out. Histological scoring methods refer to previous studies [25]. ERS-related proteins (caspase-3, GRP78, and CHOP) were detected by immunohistochemistry.

### 2.15. Statistical Analysis

SPSS 20.0 software was used to perform data analysis, and the measurement data were presented as mean ± standard deviation. Differences between two groups were analyzed by Student's *t*-test. Comparisons among multiple groups were analyzed using one-way ANOVA, and data were nonnormally distributed using the Kruskal-Wallis rank sum test. *P* < 0.05 was considered statistically significant. Graphpad prism 9.0 software and Figdraw were used to draw figures.

## 3. Results

### 3.1. ERS Level Increased with IVDD

**Figure 1:**
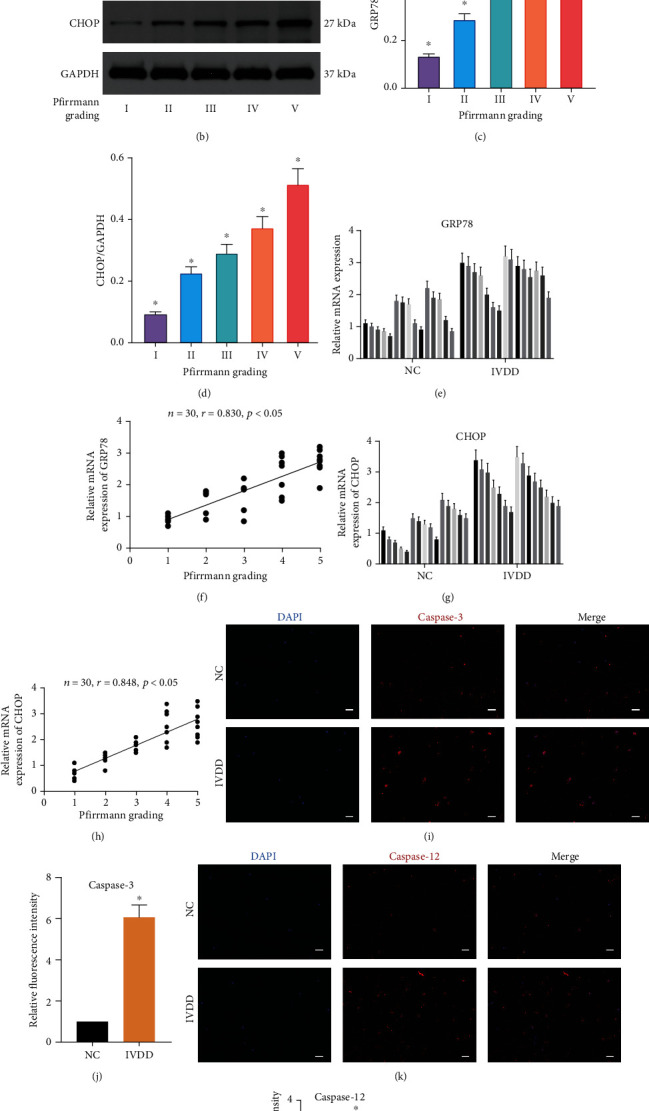
ERS level during IVDD in human NP tissues. (a) Magnetic resonance images of intervertebral discs of five different degenerative grades. (b–d) WB was used to detect the expression of ERS-related proteins in IVD of different degenerative grades, and the relative quantitative data (c, d) was calculated accordingly. ^∗^*P* < 0.05 vs. any Pfirrmann grading. (e) GRP78 mRNA level was measured by qRT-PCR in normal and degenerative NP tissues. (f) Correlation analysis between GRP78 mRNA level and Pfirrmann grading. *n* = 30. (g) CHOP mRNA level was measured by qRT-PCR in normal and degenerative NP tissues. (h) Correlation analysis between CHOP mRNA level and Pfirrmann grading. *n* = 30. (i–l) Representative images of caspase-3 (i) and caspase-12 (k) expression was detected by immunofluorescence analysis and the relative fluorescence intensity was calculated in normal and degenerative NP tissues. ^∗^*P* < 0.05 vs. NC group. Scale bar = 50 *μ*m. (Error bars showed means ± SD; *n* = 3).

We first used WB to analyze the expression of ERS-associated proteins in 5 different degenerative grades of IVD (Figure 1(a)). Both glucose-regulated protein 78 (GRP78) and C/EBP homologous protein (CHOP) increased with the degree of degeneration (Figures 1(b)–1(d)). Next, the expression of GRP78 (Figures 1(e) and 1(f)) and CHOP (Figures 1(g) and 1(h)) mRNA in normal and degenerated NP tissues was detected by qRT-PCR and correlation analysis was performed. It illustrated that the contents of both mRNAs were positively correlated with the degeneration grade. And the more severe the degeneration was, the more abundant the content was. Immunofluorescence was used to detect apoptosis-related proteins in the NP tissues of the two groups, and we found that the expression of caspase-3 (Figures 1(i) and 1(j)) and caspase-12 (Figures 1(k) and 1(l)) in the degenerated NP tissue was higher than that in the control group. These results suggested that degeneration of the NP was accompanied by more severe ERS and apoptosis.

### 3.2. SFN Alleviated AGEs-Induced ERS and Apoptosis in NPCs

**Figure 2:**
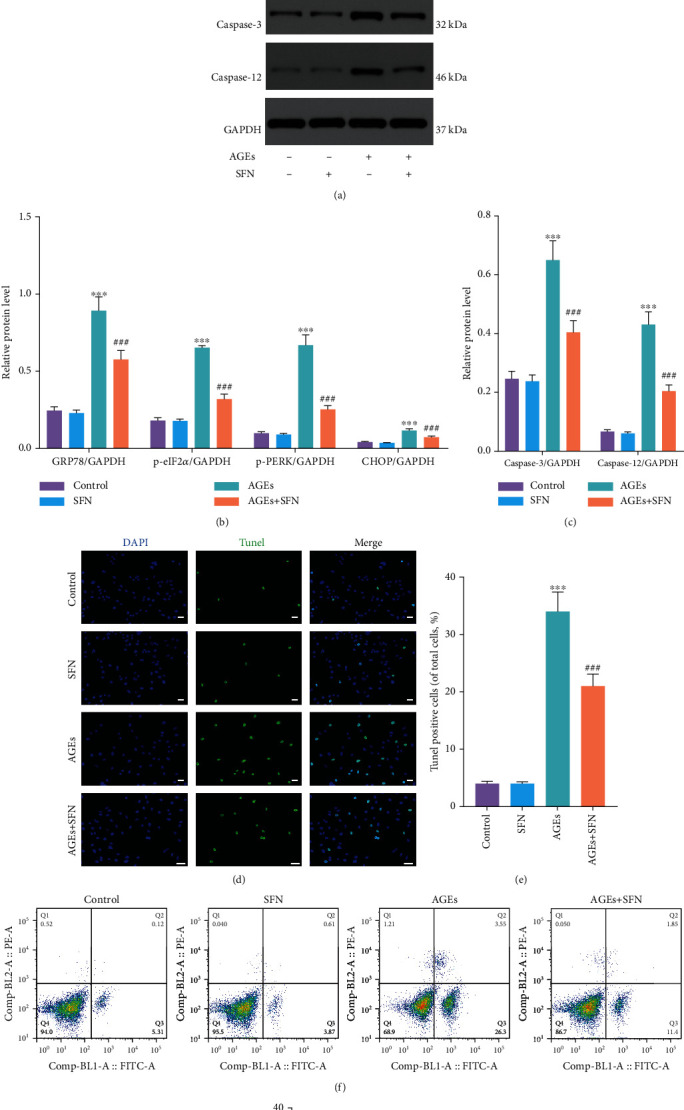
SFN alleviated ERS and apoptosis of NPCs induced by AGEs. NPCs were cultured under 1% hypoxia to simulate the hypoxia environment of intervertebral disc in vivo. (a–c) WB was used to detect the expression of ERS-related proteins and apoptosis-related proteins, and the relative quantitative data (b, c) was calculated accordingly. (d, e) TUNEL staining was used to detect the apoptosis of NPCs, and the relative quantitative data was calculated accordingly. Scale bar = 50 *μ*m. (f, g) The apoptosis of NPCs was detected by flow cytometry. (Error bars showed means ± SD; *n* = 3; ^∗^*P* < 0.05, ^∗∗^*P* < 0.01, ^∗∗∗^*P* < 0.001, vs. control group; ^#^*P* < 0.05, ^##^*P* < 0.01, ^###^*P* < 0.001, vs. AGEs group).

AGEs can lead to ERS in cells [26], and we used it to construct a cellular model of ERS. WB assays demonstrated that the expression levels of GRP78 and CHOP in AGEs treated NPCs were markedly elevated, as were the phosphorylation level of the eukaryotic translation-initiation factor 2*α* (eIF2*α*) and protein kinase R-like endoplasmic reticulum kinase (PERK). However, SFN application alleviated AGEs-induced ERS (Figures 2(a) and 2(b)). AGEs also led to increased caspase-3 and caspase-12 levels in NPCs, which could be reduced by SFN (Figures 2(a)–2(c)). TUNEL staining showed that AGEs significantly increased the number of apoptotic cells, while SFN played a protective role and reduced the proapoptotic effect of AGEs (Figures 2(d) and 2(e)). Apoptosis was further detected by flow cytometry. AGEs significantly increased the apoptosis rate of NPCs, while SFN markedly reversed this trend (Figures 2(f) and 2(g)).

### 3.3. Nrf-2 Inhibitor Mitigated the Protective Effect of SFN

**Figure 3:**
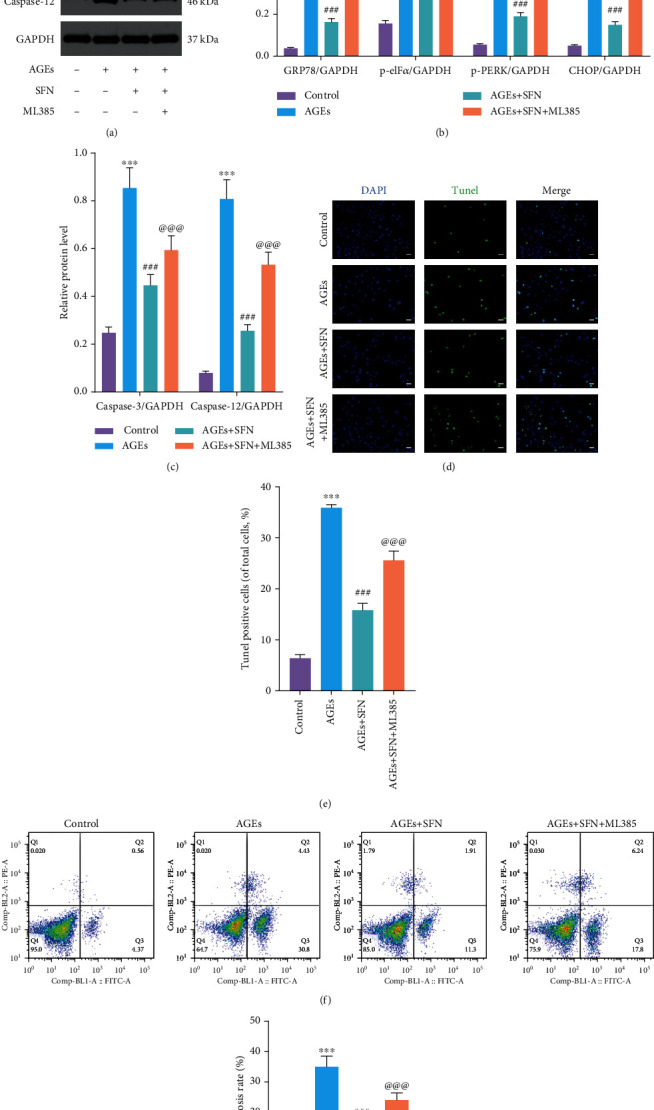
The protective effects of SFN in ameliorating ERS and reducing apoptosis in NPCs were abolished by the Nrf-2 inhibitor ML385. NPCs were cultured under 1% hypoxia to simulate the hypoxia environment of intervertebral disc in vivo. (a–c) WB was used to detect the expression of ERS-related proteins and apoptosis-related proteins, and the relative quantitative data (b, c) was calculated accordingly. (d, e) TUNEL staining was used to detect the apoptosis of NPCs, and the relative quantitative data was calculated accordingly. Scale bar = 50 *μ*m. (f, g) The apoptosis of NPCs was detected by flow cytometry. (Error bars showed means ± SD; *n* = 3; ^∗^*P* < 0.05, ^∗∗^*P* < 0.01, ^∗∗∗^*P* < 0.001, vs. control group; ^#^*P* < 0.05, ^##^*P* < 0.01, ^###^*P* < 0.001, vs. AGEs group; ^@^*P* < 0.05, ^@@^*P* < 0.01, ^@@@^*P* < 0.001, vs. AGEs+SFN group).

We further investigated whether the alleviating effect of SFN on ERS and apoptosis was through Nrf-2, and we used a Nrf-2 inhibitor ML385. When Nrf-2 was inhibited, the ability of SFN to suppress the expression of ERS-related proteins decreased and the level of ERS increased (Figures 3(a) and 3(b)). Similarly, caspase-3 and caspase-12 elevated after Nrf-2 was inhibited (Figures 3(a) and 3(c)). TUNEL staining showed that after the application of ML385, the number of TUNEL positive cells raised significantly, indicating that the number of apoptosis of NPCs increased (Figures 3(d) and 3(e)). Flow cytometry further suggested that ML385 mitigated the antiapoptotic effect of SFN (Figures 3(f) and 3(g)). These results demonstrated that SFN alleviated ERS and apoptosis by activating Nrf-2.

### 3.4. Knockdown of HO-1 Reversed the Protective Effect of SFN

**Figure 4:**
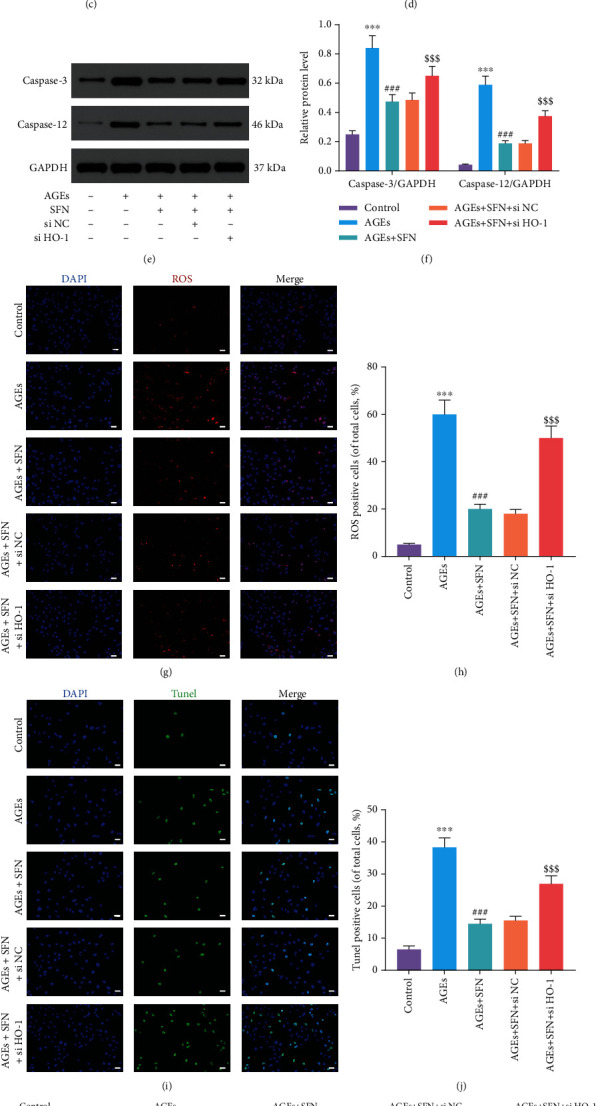
After inhibiting HO-1, the protective effect of SFN on NPC was eliminated. NPCs were cultured under 1% hypoxia to simulate the hypoxia environment of intervertebral disc in vivo. (a, b) The inhibitory effect of si-RNA on HO-1 was detected by WB. ^@@@^*P* < 0.001. (c–f) WB was used to detect the expression of (c, d) ERS-related proteins and (e, f) apoptosis-related proteins, and the relative quantitative data was calculated accordingly. (g) ROS in the NPCs was labeled with CellROX Deep Red Reagent, and the nucleus was dyed blue by DAPI. Representative images were taken by fluorescence microscope. Scale bar = 50 *μ*m. (h) Quantitative analysis of ROS level. (i, j) TUNEL staining was used to detect the apoptosis of NPCs, and the relative quantitative data was calculated accordingly. Scale bar = 50 *μ*m. (k, l) The apoptosis of NPCs was detected by flow cytometry. (Error bars showed means ± SD; *n* = 3; ^∗^*P* < 0.05, ^∗∗^*P* < 0.01, ^∗∗∗^*P* < 0.001, vs. control group; ^#^*P* < 0.05, ^##^*P* < 0.01, ^###^*P* < 0.001, vs. AGEs group; ^$^*P* < 0.05, ^$$^*P* < 0.01, ^$$$^*P* < 0.001, vs. AGEs+SFN + si NC group).

We used siRNA to knockdown HO-1. WB showed that HO-1 was successfully inhibited by siRNA (Figures 4(a) and 4(b)). When HO-1 was inhibited, the ability of SFN to alleviate ERS was substantially attenuated (Figures 4(c) and 4(d)). Meanwhile, the function of SFN on inhibiting the expression of apoptosis-associated proteins was also abolished (Figures 4(e) and 4(f)). Next, we observed ROS in NPCs by immunofluorescence. AGEs caused intracellular ROS accumulation in NPCs, which was alleviated by SFN. However, when HO-1 was inhibited, the protective effect of SFN was no longer present (Figures 4(g) and 4(h)). TUNEL staining (Figures 4(i) and 4(j)) and flow cytometry (Figures 4(k) and 4(l)) further confirmed that si HO-1 weakened the protective effect of SFN and increased the apoptosis of NPCs. The above results indicated that SFN played a role in attenuating ERS and apoptosis via HO-1.

### 3.5. SFN Promoted Nrf-2 Translocation into the Nucleus

**Figure 5:**
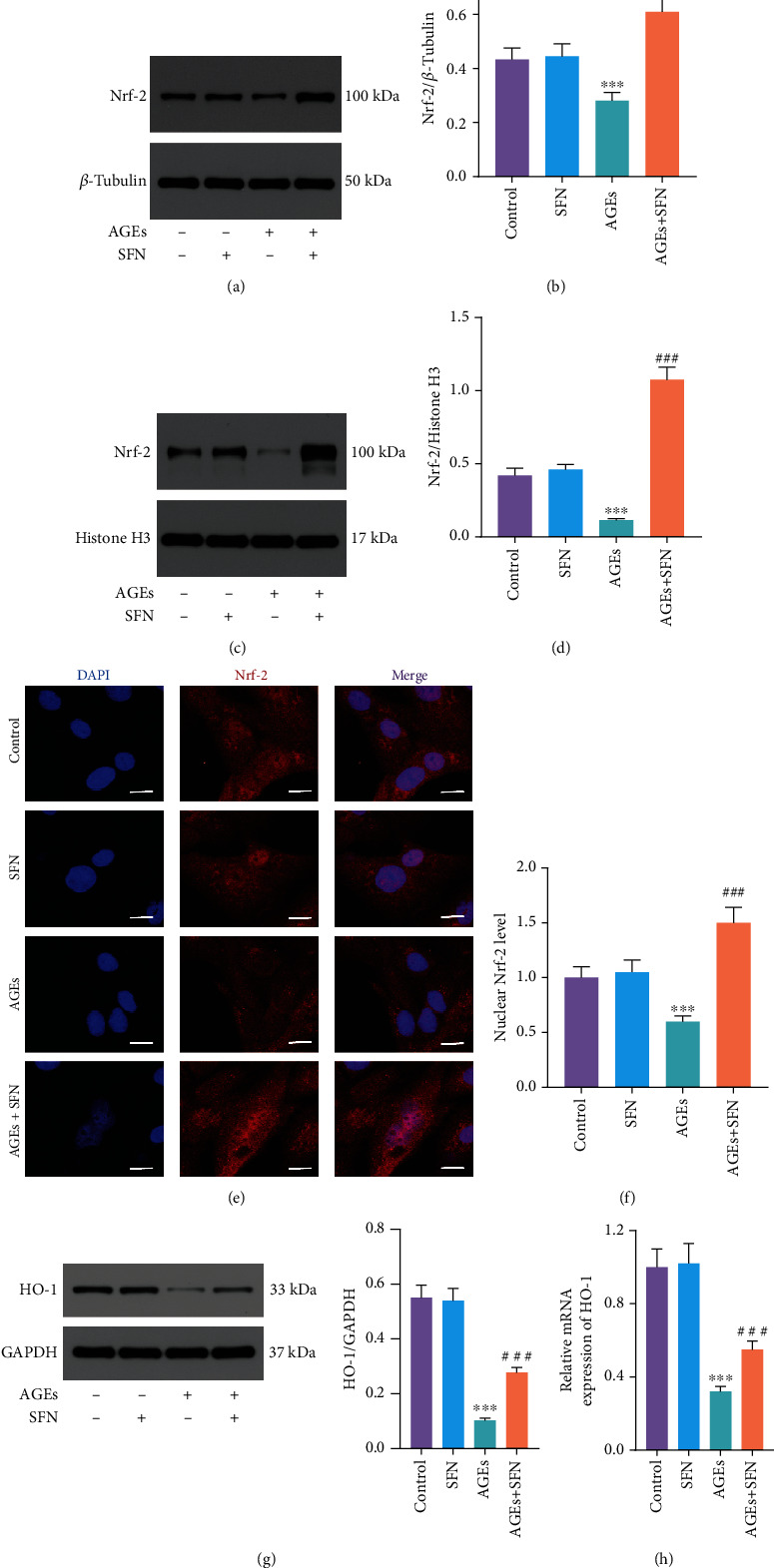
SFN promoted Nrf-2 translocation into the nucleus. (a, b) Nrf-2 expression in the cytoplasm was detected by WB. (c, d) Nrf-2 expression in the nucleus was detected by WB. (e, f) Nrf-2 fluorescence staining showed its distribution in NPCs. The nucleus was dyed blue, Nrf-2 was dyed red, and Nrf-2 in the nucleus was purple. Scale bar = 20 *μ*m. (g) WB was used to detect the expression of HO-1 protein. (h) HO-1 mRNA level was measured by qRT-PCR. (Error bars showed means ± SD; *n* = 3; ^∗^*P* < 0.05, ^∗∗^*P* < 0.01, ^∗∗∗^*P* < 0.001, vs. control group; ^#^*P* < 0.05, ^##^*P* < 0.01, ^###^*P* < 0.001, vs. AGEs group).

Since Nrf-2 plays a role in the nucleus, we further studied whether SFN can boost the translocation of Nrf-2 into the nucleus. WB showed that AGEs could decrease Nrf-2 in the cytoplasm. But when SFN was used, the level of Nrf-2 elevated (Figures 5(a) and 5(b)). Similarly, AGEs reduced Nrf-2 in the nucleus, whereas SFN application increased its level (Figures 5(c) and 5(d)). In addition, we visualized its intracellular distribution by fluorescent staining of Nrf-2 (Figures 5(e) and 5(f)). The nucleus was dyed blue, Nrf-2 was dyed red, and Nrf-2 in the nucleus was purple. In AGEs+SFN group, the purple fluorescence intensity of the nucleus increased significantly. It could be seen that SFN have the ability to promote the translocation of Nrf-2 from the cytoplasm into the nucleus. We further studied the effect of SFN on HO-1, a downstream target of Nrf-2. The results of WB and qRT-PCR showed that HO-1 increased at both the mRNA and protein levels as SFN promoted Nrf-2 to enter the nucleus (Figures 5(g) and 5(h)).

### 3.6. SFN Delayed Intervertebral Disc Degeneration In Vivo

**Figure 6:**
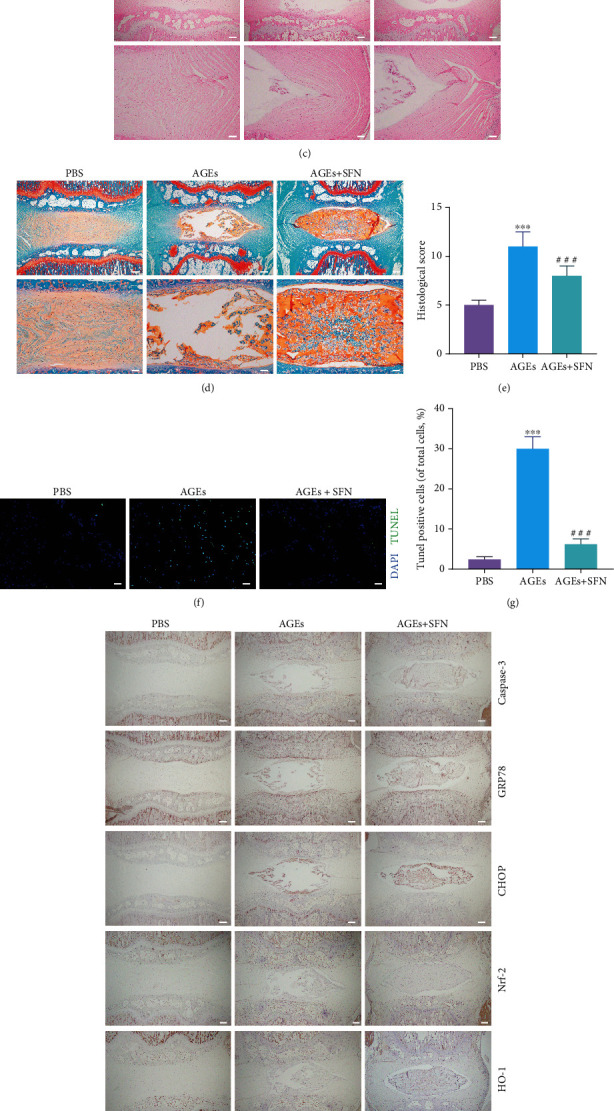
SFN delayed IVDD in vivo. (a) The tails of rats were examined by magnetic resonance at 0, 4, and 8 weeks, respectively. (b) The rat IVD were scored according to the magnetic resonance images. (c) Representative images of H&E staining of IVD. Scale bar upper = 500 *μ*m; scale bar lower = 150 *μ*m. (d) Representative images of Safranin-O/fast green staining of IVD. Scale bar upper = 500 *μ*m; scale bar lower = 150 *μ*m. (e) Histological score of IVD. (f, g) Representative images of TUNEL staining of IVD and quantification analysis were performed. Scale bar = 50 *μ*m. (h, i) Immunohistochemistry was used to detect ERS-related proteins and Nrf-2/HO-1 in the NP, and quantification analysis were performed. Scale bar = 500 *μ*m. (Error bars showed means ± SD; *n* = 10; ^∗∗∗^*P* < 0.001, vs. PBS group; ^###^P < 0.001, vs. AGEs group).

We observed the in vivo effect of SFN by injecting PBS, AGEs, and AGEs+SFN into the caudal IVD of rats. The tail of rats was examined by MR at 0, 4, and 8 weeks, respectively. The brightness and volume of IVD treated with AGEs decreased gradually with time. However, in the case of SFN cotreatment, the brightness and volume of the IVD were better maintained (Figure 6(a)). At 8 weeks, the Pfirrmann grade in the AGEs+SFN group was notably lower than that in the AGEs group (Figure 6(b)). Through H&E staining and Safranin-O/fast green staining of the IVD, it can be found that AGEs make the tissue structure of the IVD disordered or even disappeared, while SFN can maintain the tissue structure of the IVD. Moreover, the histological score of IVD in AGEs+SFN group was remarkably lower than that in AGEs group (Figures 6(c)–6(e)). TUNEL staining was used for the detection of apoptosis of NPCs. AGEs increased apoptosis of NPCs, while SFN treatment could rescue NPCs (Figures 6(f) and 6(g)). Immunohistochemistry was used to detect ERS-related proteins and Nrf-2/HO-1 in the nucleus pulposus. AGEs increased caspase-3, GRP78, and CHOP levels in the nucleus pulposus, while decresased Nrf-2 and HO-1 levels. This effect was reversed by SFN (Figures 6(h) and 6(i)). The above results suggest that SFN has in vivo effects to modulate ERS and delay disc degeneration.

## 4. Discussion

LBP affects more than half of the global population to various degrees [27]. Among the many factors that contribute to LBP, IVDD caused by genetic factors, aging, mechanical alterations, or inflammation is the most prominent trigger [28–30]. The IVD is a large fibrocartilaginous composite located between adjacent vertebral bodies and plays an extremely important role in supporting weight bearing and assisting somatic movement [31]. It consists of the annulus fibrosus, the superior and inferior cartilaginous endplates, and NP. Although the precise mechanisms of IVDD are not fully understood, accumulating evidence suggests that aberrant function of NPCs is a key contributor to IVDD [32]. Its specific manifestations are the decrease of NPCs and the increase of apoptosis [33]. At present, the main treatment strategies for LBP focus on reducing pain and other symptoms, but this will not delay IVDD [34]. Therefore, it is particularly urgent to deeply investigate the mechanisms of IVDD and thereby find treatments that have the potential to prevent or postpone IVDD.

SFN, an isothiocyanate derived from cruciferous plants, is one of the best-recognized natural products with anticancer effect, and its bioactive functions and potential application in anti-inflammation, antioxidation, anticancer, obesity, and diabetes have been extensively studied [20–22]. Oxidative stress is due to the imbalance between the generation of ROS/reactive nitrogen species (RNS) and cellular antioxidant capacity [35]. Normally, the deleterious effects of metabolically generated ROS/RNS are neutralized by antioxidant systems, and moderate level of ROS/RNS is beneficial to the normal physiological functions of cells. When the generation of ROS/RNS is beyond the bounds of antioxidant systems' scavenging ability, high concentrations of ROS/RNS cannot only react with cellular molecules such as DNA, lipids, and proteins to cause damage but also regulate intracellular signaling pathways, resulting in cell senescence and apoptosis [36].

The mechanism of cells against oxidative stress is constituted by two parts, the antioxidant enzyme system and the nonenzymatic antioxidant. Antioxidant enzymes include superoxide dismutase (SOD), glutathione-S-transferase (GST), glutathione reductase (GR), glutathione peroxidase (GP), NAD(P)H-quinone oxidoreductase 1 (NQO1), thioredoxin reductase (TR), HO-1, and others. Glutathione is the most important endogenous nonenzymatic antioxidant. These antioxidant enzymes and nonenzymatic antioxidants are regulated by the Nrf-2-related pathway [16, 17]. Kelch-like-ECH-associated protein 1 (Keap1) is a key repressor of Nrf-2 and contains several redox sensitive cysteine residues (cys151, cys273, and cys288) that play critical roles in the regulation of Nrf-2 signaling. Under normal conditions, Nrf-2 is locked in the cytoplasm by Keap1 and binds to ubiquitin ligase E3 complex. Nrf-2 is ubiquitinated and degraded by proteasome, and Keap1 is recycled and regenerated. When exposed to oxidative stress, Nrf-2 is released from Keap1 after the cysteine residue of Keap1 is oxidized or chemically modified [37, 38]. Nrf-2 translocates to the nucleus and combines with small Maf (sMAf) protein to form heterodimer. After that, Nrf-2 binds with antioxidant responsive element (ARE) located in the promoter region of many cell protective genes to activate the transcription of a series of downstream antioxidant genes. SFN can enhance Nrf-2 transcription by decreasing the methylation of the initial 15 CPGs of the Nrf-2 promoter [39]. SFN can also prevent the binding of Keap1 and Nrf-2 by chemically modifying the cysteine residues (mainly cys151) of Keap1, which in turn attenuates the ubiquitination and degradation of Nrf-2, leading to the accumulation of Nrf-2 and the enhancement of Nrf-2-regulated downstream gene transcription [37]. In this study, we found that SFN promoted the entry of Nrf-2 into the nucleus and reduced the accumulation of ROS caused by AGEs. But when HO-1 was inhibited, the alleviating effect of SFN against oxidative stress was weakened.

ER is the main organelle for protein synthesis, calcium ion storage, and lipid synthesis. Various stimuli, such as oxidative stress, will disrupt ER homeostasis, leading to UPR, misfolded protein accumulation, and pathological changes, which is ERS. Three cytoprotective mechanisms are mainly triggered by ERS [40]. Firstly, upregulated chaperone expression such as GRP78. GRP78 activates the three pathways of the UPR to assist protein refolding. Secondly, alpha subunit of eukaryotic initiation factor 2 (eIF2*α*) was phosphorylated by protein kinase R-like endoplasmic reticulum kinase (PERK), which reduces protein translation. Thirdly, protein aggregates are degraded via the ER associated degradation pathway, the ubiquitin proteasome pathway, or autophagy. However, excessive ERS for a long time will lead to the activation of inflammatory pathway NF-*κ*B, induce aging, and lead to ER specific apoptosis. ERS specific apoptosis is mediated by CHOP, which can induce the expression of a large number of proapoptotic factors including tribbles homolog 3 (TRB3), growth arrest and DNA damage-inducible protein 34 (DADD34), and death receptors 5 (DR5) [41]. In addition, ERS can activate B-cell lymphoma-2 (Bcl-2) family members, caspase-3, caspase-12, and c-Jun N-terminal kinase (JNK) to induce apoptosis [42]. In this study, a cellular model of ERS was constructed by AGEs. We found that AGEs activated the PERK pathway in ERS, and ERS significantly increased the level of apoptosis in the NPCs. SFN alleviated the negative effect of AGEs, but this therapeutic effect was abolished when Nrf-2 or HO-1 was inhibited.

Our study also had some limitations. Firstly, we only studied the effect of SFN on ERS in NPCs, ignoring its effect on other organelles. For example, Xu et al. [43] found that SFN can improve mitochondrial function in NPCs. In addition, ERS has three signaling pathways, and this study only involved PERK pathway. Finally, we only investigated the role of apoptosis in IVDD, ignoring inflammation and so on. These limitations will be improved in future research.

## 5. Conclusion

**Figure 7:**
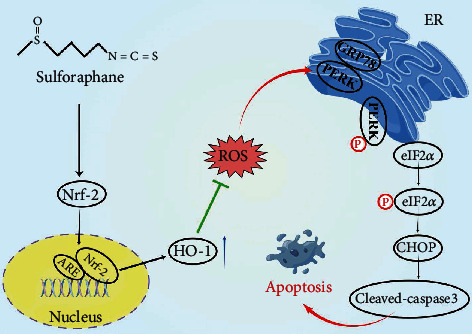
Schematic diagram of the major findings in this study. SFN promotes the entry of Nrf-2 into the nucleus and increases the expression of HO-1, thereby reducing intracellular ROS, alleviating ERS and NPCs apoptosis, and delaying IVDD.

We found that the levels of ERS-associated proteins and cell apoptosis are elevated during IVDD. SFN could mitigate ERS-induced NPCs apoptosis via Nrf-2/HO-1 pathway under the AGEs stimulation in vitro (Figure 7). Besides, the intradiscal injection of SFN could alleviate ERS-associated apoptosis during IVDD and may delay IVDD progression in vivo. This work provided a novel idea and experimental basis for the study of the mechanism and treatment of IVDD.

## Data Availability

The data used to support the findings of this study are available from the corresponding authors upon request.

## Abbreviations

IVDD: Intervertebral disc degeneration
ROS: Reactive oxygen species
NPCs: Nucleus pulposus cells
ERS: Endoplasmic reticulum stress
Nrf-2: Nuclear factor E2-related factor 2
SFN: Sulforaphane
AGEs: Advanced glycation end products
HO-1: Heme oxygenase 1
LBP: Low back pain
ECM: Extracellular matrix
UPR: Unfolded protein response
NC: Negative control
WB: Western blot
qRT-PCR: Quantitative real-time polymerase chain reaction
DAPI: 4-6-Diamidino-2-phenylindole
TUNEL: TdT-mediated dUTP nick end labeling
siRNA: Small interfering RNA
H&E: Hematoxylin and eosin
GRP78: Glucose-regulated protein 78
CHOP: C/EBP homologous protein
eIF2*α*: Eukaryotic translation-initiation factor 2*α*
PERK: Protein kinase R-like endoplasmic reticulum kinase
RNS: Reactive nitrogen species
SOD: Superoxide dismutase
GST: Glutathione-S-transferase
GR: Glutathione reductase
GP: Glutathione peroxidase
NQO1: NAD(P)H-quinone oxidoreductase 1
TR: Thioredoxin reductase
Keap1: Kelch-like-ECH-associated protein 1
ARE: Antioxidant responsive element
TRB3: Tribbles homolog 3
DADD34: Growth arrest and DNA damage-inducible protein 34
DR5: Death receptors 5
Bcl-2: B-cell lymphoma-2
JNK: C-Jun N-terminal kinase.
